# Nutrition for Older Athletes: Focus on Sex-Differences

**DOI:** 10.3390/nu13051409

**Published:** 2021-04-22

**Authors:** Barbara Strasser, Dominik Pesta, Jörn Rittweger, Johannes Burtscher, Martin Burtscher

**Affiliations:** 1 Medical Faculty, Sigmund Freud Private University, A-1020 Vienna, Austria; 2 Institute of Aerospace Medicine, German Aerospace Center (DLR), D-51147 Cologne, Germany; Dominik.Pesta@dlr.de (D.P.); joern.rittweger@dlr.de (J.R.); 3 Centre for Endocrinology, Diabetes and Preventive Medicine (CEDP), University Hospital Cologne, D-50931 Cologne, Germany; 4 Cologne Excellence Cluster on Cellular Stress Responses in Aging-Associated Diseases (CECAD), D-50931 Cologne, Germany; 5 Institute for Clinical Diabetology, German Diabetes Center, Leibniz Center for Diabetes Research at Heinrich-Heine University Düsseldorf, D-40225 Düsseldorf, Germany; 6 German Center for Diabetes Research (DZD e.V.), D-85764 Neuherberg, Germany; 7 Department of Sport Science, University of Innsbruck, A-6020 Innsbruck, Austria; Martin.Burtscher@uibk.ac.at; 8 Department of Biomedical Sciences, University of Lausanne, CH-1015 Lausanne, Switzerland; johannes.burtscher@unil.ch

**Keywords:** aging, cardiorespiratory system, exercise, hormones, masters athletes, muscle, nutrition, protein

## Abstract

Regular physical exercise and a healthy diet are major determinants of a healthy lifespan. Although aging is associated with declining endurance performance and muscle function, these components can favorably be modified by regular physical activity and especially by exercise training at all ages in both sexes. In addition, age-related changes in body composition and metabolism, which affect even highly trained masters athletes, can in part be compensated for by higher exercise metabolic efficiency in active individuals. Accordingly, masters athletes are often considered as a role model for healthy aging and their physical capacities are an impressive example of what is possible in aging individuals. In the present review, we first discuss physiological changes, performance and trainability of older athletes with a focus on sex differences. Second, we describe the most important hormonal alterations occurring during aging pertaining regulation of appetite, glucose homeostasis and energy expenditure and the modulatory role of exercise training. The third part highlights nutritional aspects that may support health and physical performance for older athletes. Key nutrition-related concerns include the need for adequate energy and protein intake for preventing low bone and muscle mass and a higher demand for specific nutrients (e.g., vitamin D and probiotics) that may reduce the infection burden in masters athletes. Fourth, we present important research findings on the association between exercise, nutrition and the microbiota, which represents a rapidly developing field in sports nutrition.

## 1. Introduction

Nutrition and physical activity (PA) are the two main modifiable factors that determine health and well-being in modern civilization. These two factors are often studied and considered as separate entities, although they are, in reality, inter-related in various ways. To give some examples of this interplay, PA can shift the nutrient spectrum that human metabolism utilizes; lack of PA leads to accumulation of ectopic fat in liver, muscle, and potentially other organs [1,2]; malnutrition hampers physical and mental performance; overfeeding leads to expansion of fat deposits, thus increasing body inertia and deteriorating physical fitness [3]. One motivation for this review, therefore, is to raise awareness of the interplay between nutrition and PA/performance for readers with either of the two backgrounds.

The topic of diet and PA becomes particularly relevant in combination with aging, as aging is generally associated with generalized inflammation and exaggerated disease burden [4]. Ample evidence suggests that regular exercise and optimized nutrition can help to reduce disease burden [5,6,7]. Therefore, the World Health Organization (WHO) has taken action to promote PA across the entire age spectrum [8], and many governments have developed national strategies also with the interest to mitigate age-related morbidity. However, older people become typically more and more sedentary with increasing age, and it is often challenging to engage them in regular physical exercise. 

**Figure 1:**
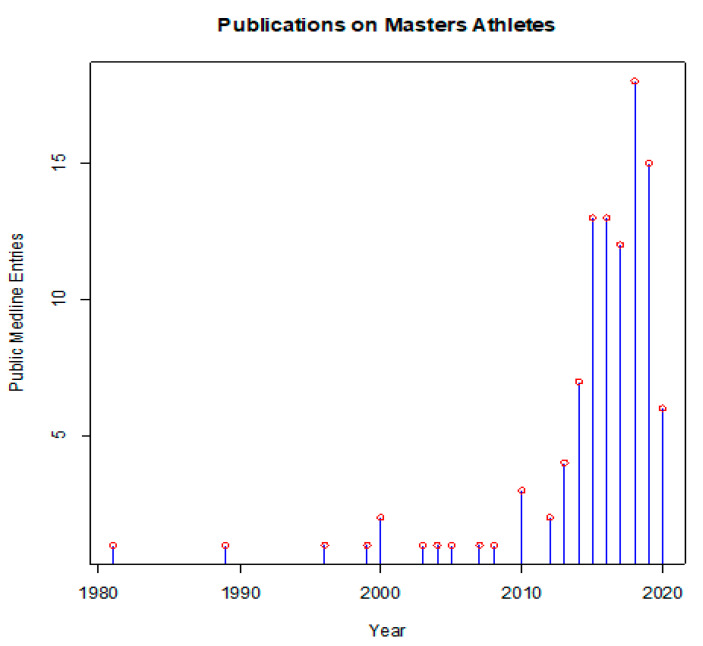
Publications on masters athletes, plotted against year of publication. Date are the result of a literature research on public Medline on 1 October 2021, using the following search terms: ((master athlete (Title/Abstract)) OR (masters athlete (Title/Abstract)) OR (veteran athlete (Title/Abstract)).

In this sense, masters athletes can offer interesting insights. These people train for and compete in athletic events beyond the typical age of retirement from sports. Many of them follow rigorous training regimens, often over decades or even their entire adult life. Beyond their intrinsic motivation to be physically active, they are driven by the desire to excel in sports, but also to maintain their health. The scientific literature has long neglected this topic, and only the past decade has seen an increasing number of publications on all aspects of masters athletics (Figure 1). 

Naturally, a plethora of questions arise with regard to the nutritional support for these aging athletes. Thus, as with any athlete, also the masters athletes wish to reduce their body fat, in order to optimize athletic performance. But what are the energetic requirements, given that resting metabolic rate (RMR) typically dwindles with age? How can the requirements for the intake of protein, vitamins and other micronutrients be met when the total intake is reduced for the sake of body composition? Which nutrients are physiologically opportune for optimized performance, and which ones should be recommended to facilitate recovery and prevention of injury? The field of nutritional support for athletes has traditionally focused on young elite athletes, and there is no clear answer to these and other questions pertinent to the topic. Hence, the present paper attempts to collate the relevant information in order to (a) provide recommendations where evidence is available, and to (b) flag the most important open question for future research.

## 2. Physiological Changes in Older Athletes

### 2.1. Oxygen Delivery and Utilization Systems

**Figure 2:**
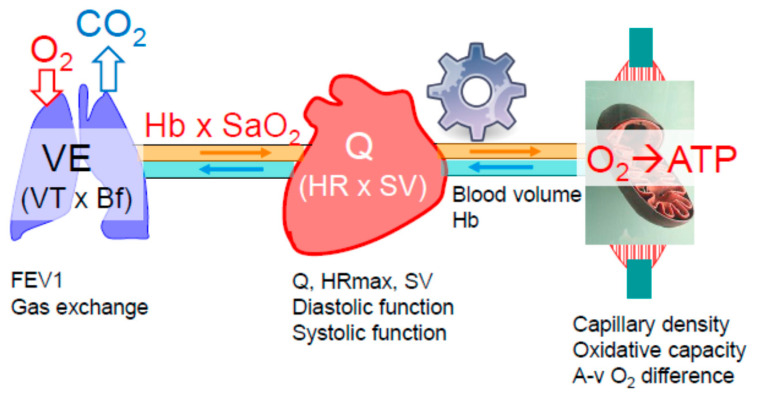
The lung–heart–muscle axis involved in oxygen delivery and utilization. Main cardiorespiratory parameters specifying organ function at rest and during exercise: minute ventilation (VE), cardiac output (Q), and oxygen extraction in the skeletal muscle. Parameters listed below the organs represent those which are primarily affected by the aging process. Oxygen, O_2_; carbon dioxide, CO_2_; tidal volume, VT; breathing frequency, Bf; hemoglobin concentration, Hb; arterial oxygen saturation, SaO_2_; heart rate, HR; adenosine triphosphate, ATP.

The exercising skeletal muscles rely on continuous oxygen supply that is precisely matched to the metabolic requirements of the exercise intensity. If, at any intensity, the oxygen supply becomes limited, anaerobic metabolism compensates for the lacking oxygen, indicated by increasing lactate concentration [9,10]. Oxygen has to be transported from the ambient air to the oxygen-respiring mitochondria of the working muscles. This is involving a series of steps, namely oxygen diffusion (driven by the existing pressure gradient) from the alveoli into the pulmonary capillary blood, the oxygenated blood (oxygen is primarily bound to hemoglobin) is pumped by the heart to the skeletal muscles, where oxygen is converted to adenosine triphosphate (ATP), providing energy to power the working muscles (Figure 2). Oxygen delivery (DO_2_) to the muscles is determined by the cardiac output (heart rate x stroke volume, Q), the hemoglobin concentration (Hb) and the level of its saturation (SaO_2_), according to the equation:

DO_2_ = Q × Hb × SaO_2_ × K

K indicates the Hüfner coefficient for hemoglobin-oxygen binding capacity which is 1.33 mL/g.

The extraction of oxygen utilized by the mitochondria is represented by the arterio-venous oxygen difference (a-v O_2_ difference). As the need for oxygen is increasing with increasing exercise intensity, minute ventilation, cardiac output, muscle perfusion and oxygen extraction all have to increase. According to the Fick principle for individual aerobic capacity, the highest achievable rate of oxygen uptake (VO_2_max) equals Q × a-v O_2_ extraction. All organs involved in oxygen transport and utilization may contribute to VO_2_max decline in the aging athlete. 

#### 2.1.1. The Pulmonary System

Although data on the breathing reserve (BR; maximal voluntary ventilation related to minute ventilation during maximal exercise) do not indicate a considerable ventilatory limitation of VO_2_max in healthy older individuals [11], it can become a limiting factor in some highly trained aged athletes [12]. In addition, diffusion capacity of the lung is predictive of marathon performance, and it decreases markedly with age, an effect from which masters athletes are not exempt [13]. Probably as a consequence of this, exercise-induced hypoxemia is more prevalent in highly fit older individuals compared to the healthy general population and fatiguing work of the respiratory muscles may provoke vasoconstriction in the leg muscles and compromise Q [14]. Thus, VO_2_max restriction by the pulmonary system depends on the level of fitness (the higher the more likely) and the age-related degree of decrease in respiratory function along with structural changes, i.e., declining respiratory muscle strength and endurance, enhanced rigidity of the chest wall, loss of elastic recoil, reduction of the alveolar surface area and the number of capillaries perfusing the lung [11,15,16]. A low BR, reduced forced expiratory volume in one second (FEV1) and exercise-induced hypoxemia are potential markers for pulmonary limitations of VO_2_max in the older athlete [17] (Figure 2).

#### 2.1.2. The Cardiovascular System

It is obvious that a precise interplay between pulmonary ventilation, DO_2_ and the extraction of oxygen by the muscle tissue is a prerequisite for properly matching oxygen need and demand. The contribution of the cardiovascular system, in particular Qmax, is considered as the main determinant of VO_2_max in young people when including large skeletal muscle groups, e.g., leg cycling, running or cross-country skiing [18,19]. The demand of oxygen by these working muscles increases tremendously (about 10 to 20-fold, depending on the individual fitness) from rest to maximal work [10]. Consequently, a large amount of oxygenated blood has to be transported to muscles driven by the pumping heart. Thus, a 72% contribution of Qmax to VO_2_max changes has been demonstrated in sedentary young and older subjects of both sexes, and the Qmax increased to 81–89% in trained young and older subjects of both sexes [18]. The age-related decrease in cardiovascular function (particularly decline of Q which is HR x SV) will considerably affect VO_2_max. HRmax decreases according to the formula (208 (beats per min)—0.7 × age) in healthy sedentary and trained subjects, probably due to the decrease in intrinsic heart rate [20]. In addition, lower SV associated with reduced left ventricular (LV) compliance (diminished diastolic function) was shown in healthy sedentary people but seems to be preserved in masters athletes [21]. Impaired ability to modulate sympathetic vasoconstrictor activity (functional sympatholysis) and a reduced exercise hyperemia are also characteristics of aging. Again, regular PA was shown to offset these impairments [22].

#### 2.1.3. Skeletal Muscle and Mitochondria

Capillarization of skeletal muscles and muscle oxidative capacity decrease likewise with aging at least in rather sedentary subjects [23,24]. However, long-term engaging in endurance sports can largely prevent the reduction in muscle capillarization and muscle oxidative enzymatic activity with aging, e.g., in 65- to 75-year-old athletes [25]. The oxidative capacity of skeletal muscles is not considered as an important limitation of VO_2_max because it exceeds the amount of oxygen consumed during whole body exercise [26]. Thus, benefits of regular training on adaptations in the skeletal muscle rather promote improved submaximal exercise performance than VO_2_max. Such adaptations include increases in capillary supply and mitochondrial key enzyme activities favoring a higher rate of fat oxidation and a concomitant reduction in the glycolytic flux, as well as a tighter control of the acid-base status [10]. Consequently, the anaerobic threshold (submaximal endurance performance) declines at a slower rate with aging (especially in trained individuals) than VO_2_max [27,28]. A noteworthy observation is the inverse relationship between mechanical efficiency and VO_2_max (shown in elite cyclers), which was attributed to variations in the amount of efficient type I and less efficient type II fibers of working muscles [29].

### 2.2. Body Composition and Metabolism

Although aging is generally associated with a loss of lean muscle mass, exercise can modulate such losses. According to population-based studies, the prevalence of sarcopenia in healthy adults aged 60 years and older is about 10% for men and 10% for women, respectively [30]. Intriguingly, in a cross-sectional study including 156 female and male masters athletes aged between 40–79 years, no individual was categorized as sarcopenic, i.e., below normal levels of muscle mass and muscle strength or performance, according to the definition of the European Working Group for Sarcopenia in Older People [31,32]. Data from cross-sectional studies indicate that lean muscle mass and muscle strength did not decline with age in individuals aged 40–81 years who trained 4 to 5 times per week [33]. These data could indicate that declines in physical function may not be related to age alone but are rather confounded by muscle disuse and decreased levels of PA in the elderly general population [34]. On the other hand, two previous cross-sectional studies based on an anthropometric assessment of lower limb muscle volume [35] in male 115 track and field masters [36] and in 54 male master weight lifters [37] indicated a volume reduction of approximately 6% per age decade. This figure has been confirmed by a recent longitudinal study in 71 track-and-field master athletes with a mean follow-up of 4.2 years that found a reduction in calf muscle cross section, as assessed with computed tomography, by 0.6% per year in men, but no significant change in women [38]. Moreover, a recent cross-sectional study in 256 track and field masters aged 35–91 years in which whole body skeletal muscle mass was assessed via bio-electrical impedance indicates a reduction by 3.2% and 2.8% per decade in women in men, respectively [39], which was equalized by a commensurate increase in fat mass. On the other hand, older athletes of 68 years showed 17% lower body fat percentage and 12% greater leg lean mass, respectively [40]. In the general population, fat-free mass is expected to decrease the 6th decade of life onwards by approximately 2% per decade in men but not in women, while both men and women gain fat mass by 7.5% per decade [41]. Thus, whilst masters athletes likely experience muscle wasting and adipose tissue accumulation, their body composition may still be better preserved than in the non-athletic counterparts.

The question arises, which factors may trigger the age-related adipose tissue accumulation. Lifelong training increased the proportion of type I muscle fibres with a concomitant decrease of carbohydrate oxidation independent of intensity level in older athletes compared to younger man [42]. While fat oxidation capacity was similar in both groups, older athletes compensated with a higher exercise metabolic efficiency [42]. While sports and recreational activity decreased to a higher extent in men than in women over a course of ~10 years, activity levels as well as baseline age were inversely related to changes in fat mass in women only [41]. Low RMR may predispose to future weight gain. As PA contributes considerably to total energy expenditure, the question is whether regular exercise can curb age-related reductions in RMR. Although it is difficult to disentangle changes in metabolic rate from alterations of body composition with aging, evidence suggests that RMR is lower in older men and women, even after adjusting for differences in body composition, waist-to-hip ratio and smoking status [43,44]. A paper, based on the cohort of 256 track and field masters athletes mentioned above, has found that the effect of age on RMR is mostly attributable to changes in body composition [45]. While fat-free mass is a main determinant of RMR, other factors that are unrelated to differences in body composition can also explain differences in RMR of young and older individuals. Although the decline of RMR also occurs in highly physically active individuals, it is associated with reductions of exercise volume and energy intake that occur with age. However, these age-related adaptations are blunted in individuals who maintain these two components at a similar level as young physically active men [46]. Also, a higher aerobic capacity is related to a higher RMR in older athletes [47]. In a sample of 65 healthy women ranging from 21–72 years, those individuals who were regularly performing endurance exercise were spared from an age-related decline in RMR assessed by indirect calorimetry compared to their sedentary counterparts [48]. This metabolic difference may in part explain lower body weight and fat mass in active, older individuals.

In summary, the decline in metabolic rate, along with simultaneous declines and inclines, respectively, in lean mass and fat mass in the elderly can only in part be ascribed to the aging process per se. Rather, these effects seem confounded by declining levels of PA and inadequate energy intake in this population. Although body composition of older athletes is considerably better than that of less physically active age-matched individuals, the age-related decline and alterations in body composition and metabolism also takes place in this group and can in part be compensated for by higher exercise metabolic efficiency.

### 2.3. Effects of Aging on the Endocrine System and Metabolic Pathways

The aging process is accompanied by several endocrine alterations, along with changes in nearly all biological systems, including body composition, functional performance and bone mass. These aging-induced effects are often confounded by other factors, such as chronic diseases or changes of dietary patterns, and malnutrition often occur concomitantly during the process of aging. This section is not intended to give a thorough overview of endocrine changes, but discusses the most important hormonal alterations occurring during aging pertaining regulation of appetite, glucose homeostasis and energy expenditure and the modulatory role of PA.

#### 2.3.1. Thyroid Hormones

The important role of thyroid hormones in determining energy expenditure and basal metabolic rate has long been recognized [49]. With aging, there is a general increase of the incidence of thyroid diseases [50]. Apart from this increase and according to several population studies, aging is associated with subclinical hypothyroidism, i.e., increased levels of thyroid-stimulating hormone (TSH) with free thyroxine (FT4) levels remaining in the normal range [51,52]. Some authors even ascribe a beneficial adaptation of physiological aging to these reduced TSH levels in the elderly by preventing excessive catabolism [53]. While the free triiodothyronine resistance index was negatively associated with aging in males, TSH levels were positively associated with age in females [54]. These results underline a possible sex-specific effect of alterations of thyroid hormones with aging.

Although endocrine effects have to be separated from alterations of body composition and PA behavior with aging, RMR is lower in older individuals, potentially even after adjusting for differences in body composition [43]. The age-related decline of RMR, however, cannot fully be ascribed to alterations of body composition or differences in thyroid hormone status [55].

#### 2.3.2. Hypothalamic Growth Hormone-Insulin-Like Growth Factor-I Axis

This axis includes the secretion of growth hormone (GH; somatotropin) from the somatotropes of the pituitary gland into the circulation, and the successive stimulation of insulin-like growth factor-1 (IGF-1). This endocrine system drives anabolic effects on protein synthesis and growth and hence plays an important role in maintaining muscle mass. In elderly individuals, GH secretion, together with IGF-1 levels decrease starting with the third decade [56]. There is also a reduction in GH releasing hormone (GHRH)-induced GH secretion, likely reflecting changes of neurotransmitter control and reduced hypothalamic GHRH synthesis related to the aging brain [57]. Regular physical exercise is thought to modulate activity of the GH-IGF-1 axis throughout the lifespan, potentially preserving muscle mass in the elderly. With regard to that, GH responses to a cycling sprint, resistance or endurance exercise bout were compared in young and middle- aged men. While resting GH concentration and objective parameters of exertion were not different between groups, GH response to exercise was greater in the young compared to their older counterparts [58]. When investigating the effect of a 12-week resistance training program on GH and testosterone secretion in young (23 years) and older (63 years) individuals, the authors found that, regardless of age, this training modality elicits GH and testosterone secretion. Response and magnitude, however, was different between the two groups [59]. Mechano growth factor (MGF), a splice variant of the IGF-1 gene, is supposed to be an important local factor promoting satellite cell proliferation in muscle. Although short-term (5-week) resistance exercise failed to increase expression of MGF in elderly as compared to young individuals [60], longer-term training of 12-weeks was still able to upregulate expression of this factor in the elderly [61]. Exercise training can stimulate the GH-IGF-1 axis as well as MGF in the elderly, albeit somewhat less so in older as compared to younger individuals. This may reflect an age-related desensitization to mechanical loading. It has to be noted that short study duration, differences in training motivation or the inability to achieve a sufficient absolute exercise intensity in the elderly as well as a sex-bias towards male study participants may bias these findings.

#### 2.3.3. Hormones Regulating Appetite and Food Intake

Above the age of 65 years, there seems to be a decrease in appetite and food intake, which predisposes to undernutrition. A negative energy balance has implications for chronic disease progression and mortality rate [62]. Hormones mediating the anorexic effect of aging include cholecystokinin (CCK), leptin, and ghrelin. CCK is a gastrointestinal peptide hormone produced by enteroendocrine cells of the duodenum that mediate satiating effects by binding to receptors in the central nervous system. Aging is associated with increased CCK concentrations as well as a greater sensitivity to the satiety-inducing effect of this hormone [63]. In line with that, whey protein ingestion resulted in greater plasma concentrations of CCK and gastric inhibitory peptide in older compared to younger individuals [64]. In addition, the increased activity of the anorexigenic hormone leptin, an adipokine derived from adipose tissue in humans as well as the reduced activity of the orexigenic hormone ghrelin, a gastrointestinal molecule derived from the stomach, seem to further reduce hunger and thereby hamper energy intake in the elderly [65,66]. Obesity is another factor that can dysregulate the endocrine role of leptin secretion from adipocytes, so that hyperleptinemia due to high fat mass fails to negatively regulate food intake, a state termed leptin resistance [67]. A recent study showed that higher fitness levels in older individuals were associated with lower leptin levels and inflammation, regardless of adiposity, suggesting a protective effect of physical fitness towards development of leptin resistance [68]. Nevertheless, the modulatory role of PA on hormones governing appetite and food intake in the elderly remains understudied. In general, individuals with higher levels of PA experience blunted satiety and amplified hunger compared to those being less physically active, likely in order to compensate for the increased PA induced energy expenditure [69]. It seems, however, that chronic exercise can affect perceptions of hunger and energy intake independent of body composition and sex. Individuals being physically active may be more sensitive to regulating energy balance by improved adjustments of energy intake and density of food [70]. The paucity of data on this subject in older athletes of both sexes, however, does not allow definitive conclusions with regard to this age group.

#### 2.3.4. Insulin, Glucose and Metabolic Pathways Mediating Glucose Homeostasis

Insulin is the most important hormone mediating control of glucose homeostasis, i.e., cellular uptake, utilisation and endogenous production. Aging is associated with deteriorating glucose homeostasis [71]. It has been suggested that, starting from the fourth decade, fasting plasma glucose increases by about 0.055 mmol/L per decade, alongside with a gradual rise of glucose concentrations obtained after 120 min of a 75 g oral glucose tolerance test [72]. Further to that, aging is a risk factor for brain insulin resistance, i.e., impaired sensitivity of central nervous pathways to insulin [73]. Interestingly, high insulin action in the brain influences long-term weight management and is associated with a favorable body fat distribution [74]. In general, insulin is secreted in a pulsatile manner. During the basal as well as insulin-stimulated state, both amplitude and number of pulses are reduced with aging [75]. This goes hand in hand with decreased effectiveness in reducing hepatic glucose output and increased liver insulin clearance [76]. Glucagon concentrations, in turn, do not seem to be affected by age [77]. However, the rise in hepatic glucose production after glucagon stimulation, i.e., hepatic sensitivity to glucagon, is increased in elderly individuals compared to their younger counterparts [78].

Seminal studies from the lab of John Holloszy and colleagues point towards the importance of physical training for maintaining glucose tolerance. The researchers studied older lean and overweight untrained as well as trained individuals compared to young untrained and trained individuals. They found that oral glucose tolerance, defined as the area under the glucose curve, was twofold poorer in the untrained older individuals as compared to the other groups. Also, the insulin response to glucose was higher in the untrained groups as compared to the older and younger trained groups [79]. These results are suggestive of reduced glucose tolerance and insulin sensitivity with aging and a counter regulatory role of lifelong exercise training.

A similar, more recent study used the glucose clamp technique to assess insulin sensitivity in younger (age range 24–47 years) and older (age range 60–75 years) athletes as well as younger and older normal-weight and obese individuals. Insulin sensitivity was highest and similar in young and older athletes, followed by normal weight individuals and lowest in obese young and old individuals [80]. This study underlines the fact that body composition and physical inactivity, but not age per se are related to the development of insulin resistance. As glucose disposal decreases over the lifespan, several factors contributing to reduced insulin action have to be considered. These include a rise in total body fat, in particular visceral fat, and reductions of caloric intake, PA and consequent decrements in lean mass or certain drugs and age-related diseases.

In this context, it is important to also scrutinize cellular mechanisms responsible for age-induced reductions in insulin-stimulated glucose uptake. In brief, upon binding to its receptor, insulin stimulates autophosphorylation of the insulin receptor, tyrosine phosphorylation of the insulin receptor substrate-1 (IRS-1) and association with phosphoinositide 3-kinase (PI3K) and subsequent phosphorylation of Akt2 at serine 473 and threonine 308 sites and AS160 phosphorylation, which finally promotes glucose transporter type 4 (GLUT4) translocation to the plasma membrane to stimulate glucose uptake [81]. Aging can selectively affect elements of this signalling cascade. For instance, Akt phosphorylation by insulin was ~40% lower in healthy, lean, elderly individuals compared to young body mass index (BMI)-, fat mass- and habitual PA-matched volunteers [82]. This was in line with a 25% reduction in insulin-stimulated rates of muscle glucose uptake. Another study found that AS160 phosphorylation under insulin-stimulated conditions was reduced, together with ~30% lower whole-body insulin sensitivity, in aged compared to young individuals [83]. Participation in an exercise training program of either strength or endurance exercise improved whole-body insulin sensitivity and abrogated impairments in AS160 phosphorylation in these individuals. Different factors have been discussed that could contribute to this reduction in insulin action in elderly individuals. Among these, a reduction in oxidative capacity [84], impaired mitochondrial metabolic flexibility during insulin stimulation [82], oxidative stress [85] or dysfunctional regulation of mitochondrial dynamics [86] may contribute to alterations of mitochondria with aging. These changes in turn promote increased deposition of ectopic lipids within the myocyte as intramyocellular lipids (IMCL). This site of lipid deposition has repeatedly been shown to be particularly detrimental to insulin action [87]. Compared to younger volunteers, IMCL content, assessed by ^1^H magnetic resonance spectroscopy, was 70% higher in elderly individuals [82]. Decrements in mitochondrial activity together with excess caloric intake promote lipid synthesis and storage and yield bioactive diacylglycerols, which are known to accumulate within the plasma membrane, where they recruit novel protein kinase C isoforms that facilitate inhibitory phosphorylation of IRS1 [81]. Ceramides, another important class of bioactive lipids, may inhibit Akt phosphorylation [81]. These intracellular processes impair the insulin signalling pathway and eventually contribute to impaired insulin-stimulated cellular glucose uptake.

In summary, aging is associated with deteriorating glucose homeostasis. While insulin secretion is diminished over the lifespan, glucagon action is only marginally affected. Besides decrements in hormones mediating control of glucose homeostasis, decreased PA, together with reduced mitochondrial function will promote ectopic lipid deposition in muscle, which further impairs insulin signalling and glucose uptake.

### 2.4. Bone and Bone Metabolism

Bone is a hard tissue that derives its tensile stiffness from the organic extracellular matrix and its stiffness in shear and compression from an inorganic calcium-phosphate compound (so-called bone apatite). Whilst the majority of the organic matrix consists of type 1 collagen (>90%), there are also other collagens as well as other protein constituents, such as proteoglycans, carboxylated osteocalcin, osteopontin and TGF-β (Transforming growth factor beta (TGF-β)). The role of these latter constituents is only just evolving and seems to be primarily related to endocrine and regulatory functions, rather than serving mechanical purposes. Four different types of cells govern biological activity in bone. Ostoeblasts lay down bone protein onto existing bone surfaces, and they also initiate mineralization. When such bone formation drifts cease, osteocytes either become dormant as bone lining cells, or they transform into osteocytes that are fully covered by bone matrix and thus remote to the bone’s surface. However, osteocytes still communicate via gap junctions with other osteocytes and lining cells, which allows trafficking of electrolytes and small organic compounds. Osteocytes are also thought to sense mechanical strains, to transfer that information into biological signals that help to adapt bone to its mechanical purposes, and also to initiate the repair of bone microdamage through the process of bone remodeling [88,89]. Osteoclasts, finally, trans-differentiate from peripheral blood mononuclear cells and have capacity to dissolve the inorganic matrix through acidification, and subsequently also the protein phase in order to resorb bone tissue.

The four types of bone cells work in co-operation with each other. Thus, the activity of osteoclasts is under the control of osteoblasts through receptor activator of NFKB ligand (RANKL), as well as under the control of osteocytes by sclerostin [90]. In addition, TGF-β from the bone matrix stimulates activation of osteoblasts [91]. The reciprocal control of osteoblastic activity by osteoclasts is less well established, but may involve compounds such as ephrinB2 and other factors [92]. These cell-cell interactions seem fundamental for the bone remodeling process [93]. Herein, osteoclastic bone resorption precedes osteoblastic bone formation, a process that is often triggered by material microdamage [94], thus helping to repair damaged bone. Modeling, on the other hand, serves the purpose of ‘shaping’ bones through formation and resorption that occurs on opposing surfaces of the bone, thus resulting in bone ‘drifts’ [95]. Thus, remodeling and modeling are two diverse processes. Whilst modeling is the main contributor to bone turn-over at young age, the bone turn-over after closure of the growth plate is mostly due to remodeling. With regards to aging, it is also noteworthy that bone turn-over is typically greater in young than in older people [96]. This is partly due to a slowing of the remodeling process, i.e., the entire cycle from activation of osteoclasts until closure of the resorption cavity, which takes approximately 90 days in young people but 120 days in old people [93]. In addition, it has been demonstrated that the ability to respond to bone microdamage becomes blunted with increasing age [97].

It is important to understand that the largest forces that bones experience emerge from regional muscle forces and impact loading, rather than from body weight per se. As a result, muscle mass and bone mass are well-adapted to each other [98]. This mutual relationship between muscle and bone is largely driven through the bone’s response to local tissue strains [99]. Thus, the mechanostat theory proposes that bone counteracts excessive deformations with enhanced bone formation, whereas sub-threshold deformations are answered by streamlining the bone’s structure [100]. In addition, evidence suggests also an independent effect of strain rate on bone accrual. There are also several signaling pathways that interact with muscle and bone. For example, bone morphogenetic protein 1 (BMP1) has been shown to positively affect muscle growth in mice [101]. TGF-β, which initially had been known for its stimulating role for osteoblasts, has now been recognized to negatively affect calcium handling within skeletal muscle, and to thereby hamper muscles electro-mechanical coupling [102]. Moreover, undercarboxylated osteocalcin from the bone enhances insulin production, cognitive functioning and muscular fuel utilization [103]. Thus, there are several compounds from bone’s non-collagen protein phase that unfold important systemic actions on skeletal muscle and athletic performance.

In addition to the mechanical determinants, bone is also under control of several endocrine systems. Both sex hormones have important effects, with the estrogens and androgens being more effective on the endocortical and periosteal surfaces, respectively [104]. From an evolutionary perspective, estrogens may serve the role of ‘packing’ bone minerals that are not mechanically required [98], so that sufficient calcium depots are available when breastfeeding taps on the calcium resources [105]. In this regard, the complete withdrawal of estrogen after menopause is a catastrophic event for the female skeleton, leading to bone loss and an incidence rate for vertebral and femoral fractures of 1% per year, respectively [106]. In female endurance athletes, the so-called female athlete triad may occur, where underfeeding is associated with amenorrhea, low bone mineral density and fractures, even at young age [107]. In addition to sex steroids, there are also other hormones impinging on bone, such as parathyroid hormone, the D-hormone and insulin-like growth factors.

Bones are responsive to exercise [108], which is primarily thought to be through mechanical effects. Rapid running and jumping elicits larger strains and strain rates than static exercises [109], and power athletes have stronger leg bones than endurance athletes [110,111,112]. The largest effects have been observed for the humerus, the structure of which can be twice as strong in tennis and baseball players in the active as compared in the passive arm [113,114]. Side-to-side differences in the legs of jumpers are much smaller than in the arm [115], which could be due to the fact that jumpers load both legs substantially during run-ups, whereas tennis players place only small loads on their passive arms.

With advancing age, bone mass is lost in the general population, a phenomenon that is much more pronounced in the spine and in the upper extremity than in the legs [116]. Bone benefits acquired through exercise at young age can still persist several decades after cessation of exercise training [117]. Conversely, when taking up an exercise activity during adulthood, i.e., after fusion of the growth plates, then it is still possible to moderately enhance bone mass and bone strength in the loaded bones, as demonstrated by observations in masters tennis players achieved in adulthood [118]. As to another question, namely what happens to masters athletes if exercise is continued across the life span, results from cross-sectional studies suggest that the bone benefits attained at young age [119] persist into old age, although they may fade away with advancing age [120]. A recent longitudinal study in 71 track and field masters with 4-year follow-up, however, demonstrates that tibia bone mass and strength can be preserved, or even increased in male power athletes, whilst male endurance athletes and female track-and-field masters lose tibia epiphyseal bone mass at a rate of approximately 5% per decade [38]. It has to be considered, however, that some of the female masters athletes in that study were aged between 50 and 60 years, i.e., within the perimenopausal decade, and that this study could not discern between the effects of aging and of menopause. It therefore seems fair to conclude that engagement in track and field events such as sprint running and jumping has potential to maintain or increase bone health into old age.

## 3. Performance and Trainability of Older Athletes

### 3.1. Performance Decline in the Aging Athlete

**Figure 3:**
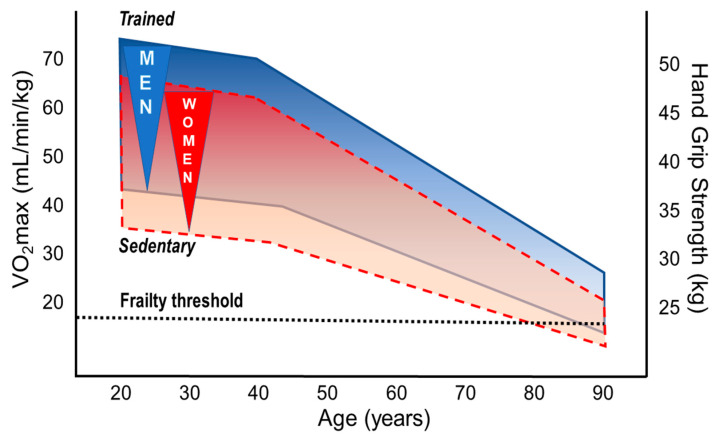
Schematic presentation of the age-related performance decline, i.e., in aerobic capacity (VO_2_max) and maximal grip strength, for both sexes, depending on physical training status, ranging from well-trained to sedentary. The figure is based on the meta-analysis from Fitzgerald et al. (1997) [130], with contributions from Burtscher et al. (2008) [28], Booth et al. (2010) [131], Landi et al. (2017) [132], and (defining frailty threshold as VO_2_max of 18 mL/min/kg) Carr et al. (2006) [133].

Declines in cardiovascular fitness and physical performance are the main features of aging human beings [121,122]. The decline starts in the third to fourth decade and depends on genetic and life-style characteristics, in particular on PA through life-span. High cardiorespiratory fitness (CRF: aerobic capacity, VO_2_max) and regular PA are associated with longevity even when adjusted for relevant confounders [122,123]. Differences of the slope in performance decline between aging athletes and their sedentary peers are still a matter of debate, but absolute performance levels remain considerably high in masters athletes of both sexes at all ages [28]. Whereas most studies primarily focused on age-dependent changes in running and swimming performance [124,125], only a few have considered a broader range of sports [126]. Data derived from mean winning performance times in track and field events of 10,000 participants in Senior Olympic Games demonstrate a slow decline from the age of 50 to 75 years which became dramatically steeper after the age of 75 [126]. Those findings have been expanded by a recent study suggesting an additional steepening of the endurance performance (in track and field events) in very old masters athletes aged 80+ [127]. Whereas no differences in performance decline in sprint versus endurance events were found for men, sprint performance declined more steeply than that of endurance in women [128]. However, when accounting for kinetic energy contributions to the metabolic costs, sprint and distance running world records depicted remarkably similar age trends [129]. A similar decline was shown in mountain runners from 50 to 70+ years; this decline was, however, slightly more pronounced in females compared to males [28]. Although the performance decline in this study seemed to be slightly steeper in masters athletes than in sedentary persons of the general population, it is important to note that endurance performance remained about 3.5 times higher in older athletes [28]. A recent review confirmed, based on current data from masters athletes, that most of the physiological mechanisms determining VO_2_max (i.e., pulmonary and cardiovascular function, blood oxygen transport capacity, skeletal muscle capillary density and oxidative capacity) are profoundly modulated by regular PA during the entire life-span [9]. In contrast to cardiovascular and skeletal muscle adaptations to PA and exercise training, pulmonary adaptations seem to play a rather minor role for the maintenance of performance in the aging athletes [9,10]. In summary, all these findings indicate that endurance performance (i.e., VO_2_max) inevitably declines when getting older but can be favorably modified by regular PA and especially by exercise training at all ages in both sexes (Figure 3).

### 3.2. Sex Differences in Performance, Decline Rates, and Training Effects

#### 3.2.1. Aerobic Fitness

Upper limits of VO_2_max values can exceed 90 mL/min/kg in young male elite athletes [134] compared to about 35 to 37 mL/min/kg in the general population aged 35–45 years [135]. Above the third age decade, VO_2_max values decline by approximately 10% per decade but this decline may be considerably modulated by training [9,10]. Extraordinarily high aerobic capacity has been reported in lifelong physically active very old individuals of both sexes. For instance, VO_2_max values of 38 (±1) vs. 21 (±1) mL/min/kg were shown for 81-year-old male endurance athletes (*n* = 9) vs. age-matched healthy untrained persons [136]. Even more impressive, a VO_2_max of 42.3 mL/min/kg has been recently reported in a 83-year old female masters runner [137]. On top, a 101-yr old athlete improved his VO_2_max following 2 years of specific training (to 103 years of age) from 31 to 35 mL/min/kg [138]. Baker and Tang compared the aging-related rates in performance decline of masters records of both sexes [126]. The age of males/females when performance had declined to 50% of the maximum performance at 30–35 years were 90/84 years for walking, 87/84 years for swimming, 78/72 years for jumping, and 74/60 years for weight lifting [126]. The larger sex differences in weight lifting compared to walking or swimming might be attributable to differences between lower and upper body performance (composition). While 12% sex difference was reported for running, swimming and cycling [139,140], the sex difference was 17% in cross country skiing (skating technique), again indicating different lower and upper body performance between sexes [135]. The relative (to body mass) VO_2_max differs by about 10% between male and female athletes, which is primarily explained by the higher percentage of body fat and lower hemoglobin levels in women [135,141]. Although this indicates similar overall effectiveness of the oxygen delivery and utilization systems in men and women, specific differences in cardiac and skeletal muscle adaptations exist. With regard to cardiac adaptation to regular exercise training, greater LV end-diastolic cavity sizes and LV mass are observed in trained versus untrained individuals, which is more pronounced in male athletes [142]. Sex differences in LV mass cannot completely be explained by the lower body size of the female athletes, but may at least partly result in the larger blood pressure increase at peak exercise in men [143]. Performance in sprint cycling (200 m) was effectively halved (from the maximum performance at 30–35 years) at an age of 80 years in males compared to an age of 59 years in female masters athletes [126]. Regular exercise training of masters athletes can prevent the shrinking of type I muscle fibers [144], and minimize loss of type II fibers, and it may also prevent fiber type grouping [145] following denervation-reinnervation cycles with aging in both sexes. Notably, females seem to develop a slower muscle fiber phenotype due to progressive slowing of discharge rates [146], likely explaining the relatively large sex difference in the decline of sprint cycling performance [126] (see below).

#### 3.2.2. Muscular Strength

**Figure 4:**
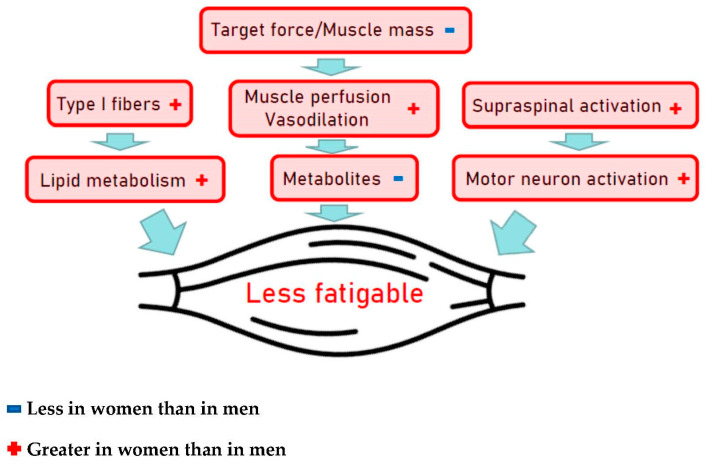
Mechanisms potentially contributing to the less fatigability of skeletal muscles in females. Modified according to Hunter [158].

Muscular strength and power are important contributors to physical fitness with an independent role in the prevention of many chronic diseases and early deaths [147]. The aging process results in decline of muscle mass and strength by about 1% per year starting in the fourth decade [148]. Peak instantaneous power declines by approximately 7% per decade when assessed via vertical jump test in endurance runners, in sprinters as well as in the general population, and very similar are reported for ergometric lower extremity power testing [36,144,149,150]. Muscle wasting, however, differs largely between individuals due to differences associated with the aging process per se but can be significantly modified by PA levels and exercise training. Most important characteristics associated with aging are the muscle architecture and fiber type composition, tendon properties and vascular control of the contracting muscle [147,151]. In athletes, the onset of declining power-lifting performance is more pronounced and progresses more rapidly than endurance performance, i.e., men’s and women’s power-lifting performance starts decreasing by 3% per year in the 4th decade and by 1% per year thereafter [152]. As shown for endurance training on cardiovascular fitness, rapid and pronounced effects of resistance training on muscle mass, muscle strength and power are well established in young and elderly individuals as well [147]. Accordingly, higher levels of muscle strength and power in the aging athletes are not surprising but seem predominantly due to hypertrophy of remaining fibers as the loss of fiber numbers seems not to be preventable by lifelong PA [153]. The magnitude of differences between sexes in muscular strength is well documented and may almost entirely be explained by the difference in muscle size of equally trained men and women [154], indicating similar muscle quality characteristics for both sexes. The overall muscle mass and power is greater in men than women and the absolute changes in muscle mass following resistance training are also larger in men, but the relative changes in strength and muscle hypertrophy are similar in both sexes [155]. From a cross-sectional study including a wide age range of men and women, better preservation of eccentric peak torque and enhanced capacity to store and utilize elastic energy with aging was shown for females compared to males [156]. While eccentric actions in a hypertrophy-targeted resistance training seem to be slightly more effective than concentric actions, both types of training should be included [157]. Skeletal muscles of males compared to females are generally stronger and more powerful, but muscles of males might be more easily fatigable. While those sex differences are primarily caused by differences in contractile mechanisms, other mechanisms, e.g., muscle perfusion, voluntary activation, etc., also represent contributing factors (Figure 4) [158].

#### 3.2.3. Mitochondria

Mitochondria are highly adaptive organelles and dynamically respond to environmental stimuli, such as nutritional states and physical exercise. Growth of mitochondrial mass, achieved by mitochondrial biogenesis, is regulated by a number of molecules including the co-transcriptional factor peroxisome-proliferator-activated receptor γ co-activator-1α (PGC-1α) [159]. Conversely, mitochondrial mass can be reduced by mitophagy, a process contributing to clearance of defective mitochondria and thus to mitochondrial quality control. Mitochondrial numbers are, furthermore, regulated by mitochondrial dynamics (i.e., fusion and fission) [160] that do not necessarily change mitochondrial mass. Physical activity is known to boost mitochondrial biogenesis and turnover, as well as respiration [161] and mitochondrial quality is positively associated with physical performance [162,163,164]. AMP-activated protein kinase (AMPK) regulates PGC-1α [165] and mitochondrial quality control [166] and is therefore an important mediator of mitochondrial biogenesis by PA. Mitochondrial functions, such as for example ATP production, are compromised in advanced age [167]. Accordingly, aging is also associated with oxidative stress and with inflammation [168], as well as with impaired mitochondrial biogenesis. The latter is at least in part mediated by reduced AMPK activity with increasing age [169], an effect that can be prominently attenuated in the skeletal muscle by exercise [170].

Although sex dimorphisms of mitochondria have been described, they are still a matter of debate and the functional consequences are not well understood. In aged rat brain a higher mitochondrial mass has been reported in males, while females had more efficient mitochondria with a better redox balance, which was in line with reduced levels of uncoupling proteins (UCP4 and UCP5) that are implicated in oxidative stress reduction [165]. This sexual mitochondrial dimorphism may be associated with higher life span and protection from some age-related neurological diseases [171]. Similarly, more efficient mitochondria in female rats have been described in the liver [172,173], cardiac muscle [174] and skeletal muscle [175]. A review of sex-related differences in mitochondria by Ventura-Clapier and colleagues [176] provides an overview on several further rodent studies in which mitochondria of female in most tissues appear to be more efficient and less affected by oxidative stress as compared to males [176]. On the other hand, the transcription of mitochondrial biogenesis-related factors has been reported to be tissue-specifically different in mice; with no differences in the liver but higher levels of related RNAs in brain and kidney of male as compared to female mice [177]. In humans, mitochondrial biogenesis could be higher in blood of females than in blood of men [178]. Conversely, ATP production rates in skeletal muscles have been observed to be lower in women than in men in another study [179].

**Figure 5:**
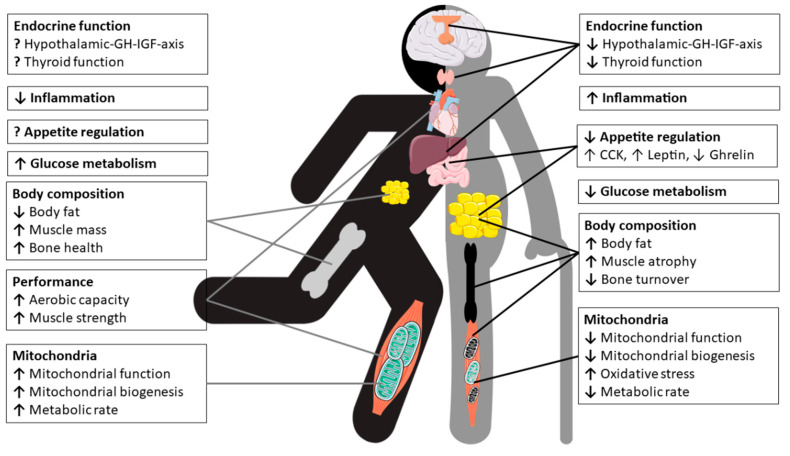
Pleiotropic effects of physical exercise and nutrition on healthy aging. The right side depicts effects of aging on endocrine function, appetite regulation, glucose metabolism, body composition and mitochondria. The left side illustrates how physical exercise can potentially mitigate effects of aging on the human body of masters athletes. **↑**, positive change; **↓**, negative change; ?, unknown effect. Cholecystokinine, CCK; growth hormone, GH; insulin-like growth factor, IGF.

In conclusion, the sex dimorphism of mitochondria appears to vary between cell types, tissues and species, as well as with age and health or physical fitness status. To better understand sex-related differences in terms of for example mitochondrial ATP production, reactive oxygen species (ROS)-production/oxidative stress or mitochondrial biogenesis and in particular functional consequences require more detailed research. However, the importance of mitochondrial integrity and efficiency for human health is well established and mitochondrial quality and biogenesis can be enhanced, e.g., by PA, in both men and women [180,181,182]. Figure 5 depicts the pleiotropc effects of physical exercise and nutrition on physiology of master’s athletes.

## 4. Nutritional Considerations for Masters Athletes

Masters athletes are often considered as a role model for successful aging and their physical capacities provide useful insight into strategies for healthy aging [183]. Although training is the primary stimulus for exercise-induced adaptations, nutrition can have a major impact on the physiological adaptations that result from exercise training and competition. However, nutrition for the older athletes needs to consider the physiological and diet-related challenges associated with aging and exercise (e.g., changing gut function and nutrient requirements with age) that affect training capacity or nutrient absorption. The most important challenges that masters athletes may face to stay competitive is, first, the maintenance of energy balance, including the risk of low energy availability and, second, anabolic resistance, where the synthetic response to muscle contraction and/or protein ingestion is blunted. For example, a protein-energy deficit can quickly lead to a loss of muscle mass, strength and function together with a transient depression of immune function so that exercise performance is compromised [184]. Furthermore, changes in body composition and hormones in the andropausal/menopausal transition can influence both muscle and bone. The aging muscle is a significant predictor for falls and fractures. Immobilization of a limb due to injury results in a sudden and dramatic muscle wasting and bone loss in conjunction with an inflammatory response, both of which may have detrimental metabolic and functional consequences. This section therefore highlights nutritional aspects that may support health and physical performance for older athletes. Key nutrition-related concerns include the need for adequate energy and protein intake for preventing low bone and muscle mass and a higher demand for specific nutrients (e.g., vitamin D and probiotics) that may reduce the inflammatory burden in masters athletes. In older adults, gut microbiota composition may represent a marker of health status and probably a predictor of functional decline. With this review, we highlight important research findings on the association between exercise, nutrition and the microbiota, which represents a rapidly developing field in sports nutrition.

### 4.1. Dietary Protein and Energy Requirements

It is well known that a balanced diet that provides enough energy to allow physical exercise is of utmost importance to stay healthy, and this is a yet more crucial factor for athletes with specific dietary needs. In sports nutrition, energy availability is defined as the energy available to promote good health once the energy cost of exercise is deducted from energy intake, relative to an athlete’s fat-free mass. Low energy availability (<30 kcal/kg of lean body mass/day) is associated with a number of disorders seen in both female and male athletes, including reduced metabolic rate, hormonal changes (e.g., satiety hormones, reproductive hormones, GH and IGF-1), poor bone health and impairments of muscle protein synthesis, immune health and performance [185,186]. Females may have special or different needs due to differences in body size and nutritional status (e.g., energy availability or iron status) as well as due to fluctuations in sex steroid hormones, for example via menopause [187]. Furthermore, physiological changes associated with aging per se such as a gradual decrease in lean body mass and subsequently in RMR, loss of appetite, changes in the composition and function of the gut microbiota but also diminished salivary secretion may increase the risk of inadequate energy intake and might require modification of the master athletes’ diet [188]. Although regular PA may lower the risk of inadequate energy intake and has the potential to maintain muscle mass and RMR with aging [46,48], recent findings including athletic populations suggest that masters athletes are still at risk of nutritional deficit [189]. In this study in master triathletes, post-exercise energy and protein intakes relative to body mass were significantly lower than the recommended dietary allowance (RDA) for younger athletes, with −40% for energy (22.7 kJ/kg), and −25% for protein (19.6 g), which may affect post-exercise recovery. Furthermore, dietary analysis revealed that female masters athletes in particular consumed significantly less carbohydrates (0.7 g/kg) post-exercise than recommended (1.0 to 1.2 g/kg).

#### 4.1.1. Optimizing Post-Exercise Recovery

Carbohydrates provide key substrates for the muscle and central nervous system during rest and exercise. It is still unclear if the recommended carbohydrate intake of 1.2 g/kg/h [190] is sufficient for this, or if a surplus could enhance post-exercise muscle glycogen resynthesis and whether female athletes or older individuals are affected differently. Until now, there is no evidence that post-exercise carbohydrate fuelling recommendations differ between ages and sexes. Thus, it is likely that carbohydrate loading strategies act in the same way in masters athletes, provided that they consume at least 8.0 g/kg body mass/day for daily fuel needs and recovery [190], additionally 30 to 70 g carbohydrates per hour of exercise—depending on exercise intensity and duration—for immune system recovery after intense exercise boots [191].

Proteins are essential nutrients for recovery from exercise. Post-exercise protein ingestion stimulates muscle protein synthesis (MPS), and the balance between synthesis and breakdown of muscle proteins determines recovery and adaptation to the exercise stimulus. Thus, sufficient provision of amino acids is critical to build muscle mass and strength with exercise training, as well as enhancing other adaptations [192]. In particular resistance exercise augments the ability of muscle to respond to protein intake. The optimal daily dietary protein intake depends on many factors, including age, sex, body size, habitual energy and nutrient intake as well as habitual exercise and PA. Moreover, acute dietary factors such as the quality of protein, defined by the spectrum of amino acids contained, the amount of protein ingested and the timing of protein ingestion and also the intake of other nutrients can influence the response of muscle to protein intake [193]. It is clear that the essential amino acids are critical for optimal stimulation of MPS, but also as signals to stimulate the process. The essential amino acid leucine is of particular interest as it is a powerful signal for stimulation of the mammalian target of rapamycin (mTORC) 1 pathway, which is responsible for the initiation of protein translation and is thus often used as a proxy measure for MPS [194]. The recommended protein intake for professional athletes is between 20–25 g of high-quality protein consumed after exercise [195], preferably through the ingestion of whole foods [196], which are rich in dietary protein, vitamins, minerals, and other macronutrients (e.g., whole milk or eggs). Recent data suggest that this amount may be suboptimal and insufficient for older people [197]. Thus, dietary protein recommendations for older adults should be increased (i.e., ≥30 g/meal or ≥1.2 g/kg body mass/day), which contain higher amounts of leucine (i.e., 78.5 mg/kg body mass/day) than current recommendations [198], and also considered on a meal-by-meal (every 3–4 h) basis [199,200]. Experts even suggested higher protein intakes for masters athletes (35–40 g/meal) to meet a daily target of ~1.5–1.6 g/kg body mass/day to optimize lean mass gains during resistance training [201]. Protein intakes in female masters athletes with energy intakes less than 1800 kcal/day are likely to be too low. To preserve lean tissue during periods of energy restriction, protein requirements are greater (i.e., 1.6–2.4 g/kg body mass/day) than during periods of energy balance [202]. Vegetarian athletes can increase the muscle anabolic potential by blending animal and plant protein sources [203], and vegan athletes should combine various plant-based proteins in a 50/50 ratio to provide a more balanced amino acid profile (e.g., maize/soybean, rice/soybean, rice/pea) or by fortifying plant-based proteins with leucine (3 g/meal) [204,205].

#### 4.1.2. Mastering Anabolic Resistance

At old age, the stimulating effects of exercise on MPS become blunted, which is referred to as anabolic resistance [206]. It was also recently demonstrated that masters triathletes aged >50 years display lower MPS rates following a bout of downhill running than younger triathletes, suggesting slower acute recovery with aging [207]. However, the latter study was not designed to address the impact of chronic exercise training on muscle anabolism with aging. The only study to date to investigate MPS in masters athletes compared to untrained older individuals reported that endurance-trained masters athletes, with an average of ~50 years of continuous training, do not display an elevated capacity to upregulate intramuscular signalling and integrated myofibrillar protein synthesis in response to unaccustomed resistance exercise training [208]. This is somewhat surprising, since masters athletes typically display superior physiological function and indices of muscle morphology compared with healthy untrained older individuals (see above, [209]). Obviously, lifelong exercise is the best approach to achieve whole-body health, but even starting later on in life will help delay age-related muscle weakness and physical disability.

There is a great interest how modifiable factors, such as diet and PA, can modulate the rate of age-related muscle loss. Stable isotope approaches revealed that the older muscle displays a reduced responsiveness to anabolic properties of amino acid feeding [210,211]. Older women in particular exhibit a blunted MPS response to feeding [206]. This anabolic resistance is now widely believed to be a key factor responsible for age-related muscle loss [212]. However, performing exercise in close temporal proximity to protein ingestion, and increasing the amount of protein ingested per meal (see above) can—at least to some extent—overcome anabolic resistance [213,214]. In support of this, cross sectional data show that senior athletes who consume protein modestly above the RDA experience higher muscle strength and quality than those consuming the RDA [215]. Moreover, supplementing with a daily dose of ∼3.5 g omega-3 fatty acids has been shown to stimulate MPS and may improve muscle mass and function in healthy older adults [216,217].

### 4.2. Bone Health and Injury Recovery

In addition to its mechanical susceptibility, bone is also a nutritionally modulated tissue and nutritional inadequacies are a risk factor for low bone mass in athletic individuals [218]. What is less clear is the influence of feeding practices on the bone response to intense exercise and training, and the current knowledge is well covered in the recent review by Sale et al. [219]. Evidence suggests that an energy availability >30 kcal/kg of lean body mass/day minimize negative effects on the bone and an energy availability of 45 kcal/kg of lean body mass/day is optimal to support bone health in the athlete [220]. This requirement is particular important to prevent the female athlete triad and to thereby prevent fatigue fractures [107]. Apart from energy availability, low carbohydrate availability negatively affects the bone, while consuming carbohydrate before, during or after exercise attenuates bone resorption to intense exercise and training in the athlete [221]. Masters athletes require protein intakes higher than the RDA (between 1.2 and 1.6 up to 2.2 g/kg body mass/day) through its support for muscle mass and function [201], but also via the increase in circulating hormones and growth factors, such as IGF-1, which have an anabolic effect on bone [222]. Nevertheless, it seems unlikely that a diet high in animal protein (∼2 g/kg) is harmful for bone health, provided that dietary calcium intake is adequate [223]. Findings even indicate beneficial effects of animal protein sources on bone strength in older adults with exercise training [224]. In addition, diets high in animal protein appear to protect against bone loss during periods of weight loss [225]. Fermented dairy products, in particular, exert beneficial effects on bone growth and mineralization, attenuation of bone loss, and reduce fracture risk [226]. Perhaps more attention should be paid to increasing fruit and vegetable intake in older athletes, because of their potassium alkali salts that the body metabolizes to bicarbonate [227,228], rather than reducing animal protein sources. An important direct or indirect mediator of bone and skeletal health is vitamin D. Vitamin D is mainly obtained through sunlight ultraviolet-B exposure (UVB) of the skin, with a small amount typically coming from the diet. It is now clear that vitamin D has important roles beyond its well-known effects on calcium and bone homeostasis. Vitamin D deficiency and insufficiency are common in athletes [229] and associated with a greater risk of low bone mass and bone injuries, such as fatigue fractures [230], which appeared to be protected by calcium (2000 mg/day) and vitamin D (800 IU/day) supplementation [231]. Higher doses with at least 1500–2000 IU/day vitamin D are required in athletes with insufficient status (circulating 25(OH)D < 40 nmol/L) [232].

#### 4.2.1. Muscle Disuse Atrophy

Skeletal muscle injuries account for over 40% of all sports-related injuries, with a two times higher risk in male than female athletes [233]. Fatigue fractures are the most common bone injuries in athletes (0.7% to 20% of all injuries), especially in women with reduced energy availability [218], and often occur during periods of high-volume and high-intensity training that are characterized by excessive and rapid increases in training and competition load [234]. Fatigue fractures emerge from prolonged mechanical overuse, so that bone’s capacity to repair microdamage through the remodelling process is overwhelmed by the emergence of new bone microdamage sites, which ultimately results in bone material fatigue [94]. Thus, the factors that predispose to fatigue fractures include the intensity, frequency and duration of loading exercises, as well as short recovery periods between loading cycles [235]. Based on the current epidemiological evidence, the masters athlete’s age, per se, does not increase the prevalence and risk of injury within competition [236], although little is known about injuries during training, including the incidence of fatigue fractures. It is rather that inadequate energy intake and/or deficits in muscle strength, flexibility or aerobic fitness increases the risk of sustained sports injuries.

Muscle disuse due to prolonged best rest (e.g., hospitalization, recovery from surgery), limb immobilization or reduced PA due to illness result in rapid muscle atrophy (∼0.5% of muscle mass per day of immobilization) and deconditioning of muscle tissue [237,238]. Furthermore, short-term (5 days) muscle disuse has been demonstrated to lower post-absorptive and post-prandial MPS rates and to induce anabolic resistance to protein ingestion in healthy young adults [239]. Thus, muscle disuse represents a unique metabolic and nutritional challenge for the young but even more so for the masters athlete as energy and macronutrient requirements are altered considerably. Although robust data on the consequences of these changing metabolic demands and the efficacy of nutritional interventions in injured athletes are still lacking, models of muscle disuse (i.e., bed-rest, step reduction) have revealed insights into the likelihood for deleterious metabolic adaptations that occur during a short-term reduction in PA with increased sedentary behavior [2,240,241]. A recent study in young adults with a habitually active lifestyle (>10,000 steps/day) provided direct evidence of a number of unfavourable adaptations to body composition with loss of lean mass (∼0.3 kg) and accretion of abdominal and liver fat, with development of whole-body insulin resistance, after 2 weeks of physical inactivity (>80% step reduction) [2]. Older adults with lower baseline lean mass and slower rate of recovery following physical inactivity may be even more prone to these acute periods of muscle disuse when compared to young people [2,241].

#### 4.2.2. Role of Nutrition for Injury Recovery

While sports nutrition has typically focused on augmenting performance and adaptations, far less attention has been given to nutrition for the injured athlete. Injury, per se, results in significant stress response and increases energy expenditure by 15% to 50%, depending on the type and severity of the injury. During the first stage following an injury, an inflammatory response is initiated accompanied by an increase in catabolic hormones (i.e., cortisol and catecholamines) and a decrease in anabolic hormones (i.e., GH, testosterone), resulting in a catabolic environment that can lead to a sudden and large loss of lean body mass [242].

Nutritional strategies have been proposed to help improve recovery from exercise-induced injuries involving immobilization and/or reduced activity [243,244,245,246,247]. One of the key considerations during the injury is to ensure that sufficient energy is consumed to prevent excessive muscle disuse atrophy and to support repair, without significantly increasing body fat. Hence, identifying energy needs during injury via indirect calorimetry or estimated using predictive equations is an important first step to maintain the caloric balance. Thereby, total daily energy expenditure can be calculated as: resting metabolic rate x stress factor (bone fracture, minor surgery = 1.2) × activity coefficient (sedentary = 1.2, lightly active = 1.4) [245]. Especially in the older athlete, where the muscle could develop anabolic resistance, it is crucial to ensure a higher protein intake for repair. The recommendation is 1.6–2.5 g/kg body mass, evenly spread across the day, every 3–4 h around a rehabilitation session, and before sleep, in amounts of 20–35 g, which contain high amounts of leucine (2.5–3 g), and additionally casein prior to sleep [246,248,249]. Other nutrients, such as creatine monohydrate (10 g/day for 2 weeks), fish oil-derived omega-3 fatty acids (4 g/day), and ß-hydroxy-ß-methylbutyrate (3 g/day) have been proposed as beneficial for the treatment of injury [248]. However, although dietary-supplement strategies may be useful if caloric intake and appetite is reduced, nutritional considerations to promote injury recovery should be explored in a food first approach, rather than a reliance on supplements [243].

### 4.3. Immune Function and Risk of Infection

Age-related declines of both the innate as well as the adaptive immune system contribute to the increased susceptibility of older individuals to acute and chronic infections, autoimmune diseases, and systemic inflammatory diseases. While a lack of PA, decreased muscle mass, and poor nutritional status facilitate immunosenescence (namely, a decline of naïve T cells and the CD4/CD8 T-cell ratio, an increase in memory/effector T-cells and senescent/exhausted T-cells) and inflammaging (characterized by elevated levels of IL-6, TNF-α, and IL-1β), moderate exercise training positively affects the composition of the T cell compartment, the function of various leukocyte subpopulations, and counteracts hallmarks of immunosenescence [250]. Thus, maintaining regular PA throughout life helps to maintain function of the immune system during aging, which could play a role in preventing infection. A recent study in masters cyclists aged 55 to 80 years found that compared with inactive older adults, the cyclists showed reduced evidence of a decline in thymic output and inflammaging [251]. Masters cyclists showed higher serum levels of the thymo-protective cytokine IL-7 and lower IL-6, which promotes thymic atrophy. In addition, maintaining high levels of aerobic fitness during aging may help prevent the accumulation of senescent T-cells [252]. While moderate exercise reduces the risk of illness [253], prolonged intense exercise is associated with a transient depression of immune function and can lead to immune impairment in athletes associated with an increased susceptibility to infections (mainly of the upper respiratory tract) [254]. There has been much interest in how dietary strategies can improve immunity in athletes. So far, there is limited evidence that the dietary practices of athletes such as low energy or carbohydrate availability suppress immunity. Athletes are recommended to follow a balanced diet to avoid a nutrient deficiencies required for proper immune function [255]. A metabolomics approach offered by Nieman and Mitmesser [191] highlights the potential impact of high carbohydrate-polyphenol food sources to counter post-exercise inflammation and to enhance oxidative and anti-viral capacity, with reduced upper respiratory illness (URI) rates [256]. Another intervention by which the immunomodulatory effects of high-intensity training might be countered is by manipulating dietary protein intake. For example, a reduced incidence of URI was observed in elite cyclists undertaking 2 weeks of high-intensity exercise training while consuming a high protein diet (3 g/kg body mass/day) [257]. The authors concluded that it is possible that immune surveillance might be maintained during heavy training by consuming a high protein diet. The new theoretical perspective offered by Walsh et al. [258] sharpens the focus on tolerogenic nutritional supplements (e.g., probiotics, vitamin C and vitamin D) shown to reduce the burden of infection in athletes at a non-damaging level. In the present section, we focus on vitamin D and probiotics, with a particular interest in the gut microbiota because of the close relationship between the microbiome and the immune system.

#### 4.3.1. Anti-Inflammatory Vitamin D

There has been increasing interest in the benefits of supplementing vitamin D during the winter months as studies demonstrate vitamin D insufficiency (25(OH)D < 50 nmol/L) in more than half of all athletes living at northerly latitudes [259]. It is important to avoid vitamin D deficiency (25(OH)D < 30 nmol/L) in order to maintain immunity and prevent URI, particularly in settings where profound vitamin D deficiency is common [260]. URI tend to be more prevalent in female endurance athletes when engaging in similarly high training loads than their male counterparts (~11 h/week of moderate-vigorous activity) [261,262]. Evidence supports an optimal circulating 25(OH)D of 75 nmol/L to prevent URI in athletes and military personnel and, furthermore, to enhance immune function and to induce anti-inflammatory actions through the induction of regulatory T cells and the inhibition of pro-inflammatory cytokine production [263]. Reducing inflammation is a key mechanism that can improve age-related skeletal muscle changes through either direct catabolic effects or indirect mechanisms (e.g., higher GH and IGF-1 concentrations, less anorexia) [264]. A recent study in sarcopenic older adults participating in a 12-week controlled resistance training program found a significant beneficial effect of daily supplementation with whey protein (22 g), essential amino acids (including 4 g leucine), and vitamin D (100 IU) compared to placebo, with a gain of 1.7 kg in fat free mass, significant decreases in C-reactive protein concentrations, and significant increases in IGF-I concentrations, accompanied by a reduced risk of malnutrition [265]. Although the authors were not able to assess the effects of vitamin D supplementation separately from essential amino acid supplementation, this study suggests that whey protein, essential amino acid and vitamin D supplementation, together with resistance training, can reduce inflammatory markers and improve anabolic markers in sarcopenic elderly. Interestingly, long-term supplementation with calcium-vitamin D fortified milk may negate these benefits because of the concomitant gains in fat mass [266]. Despite the decline in vitamin D production with aging, vitamin D sufficiency can be achieved in older athletes by regular sunlight exposure in the summer and daily 1000 IU vitamin D3 supplementation in the winter months [267].

#### 4.3.2. Gut Microbiota and Probiotics

The concept of a healthy resilient gut microbiome relies on its high richness and biodiversity [268]. The intestinal microbiota plays an important role in many metabolic processes that are beneficial to the host such as synthesis of vitamins and production of short chain fatty acids (SCFA), but also in the development of the mucosal immune system [269]. On the other hand, it has also been associated with chronic inflammation resulting from impairment of mucosal barrier, thereby contributing to the development of inflammatory, autoimmune and metabolic diseases [270]. Lower bacterial diversity has been observed with advanced age, and accumulating evidence suggests that the gut microbiota is linked to health status in aging [271]. Diet, particularly protein intake, and exercise can modify the composition, diversity and metabolic capacity of the gut microbiota [272,273,274] and may, thus, provide a practical means of enhancing gut and systemic immune function. It has been reported that exercise-induced changes in the microbiota (e.g., increased butyrate-regulating bacterial taxa and microbiome SCFA-producing capacity) are more substantial in lean versus obese adults and are largely reversed once exercise training discontinued [274], suggesting an association between microbial diversity, body composition, and physical function. Cardiorespiratory fitness (VO_2_max) has been observed to correlate with gut microbial diversity and fecal butyrate, a SCFA associated with gut health, in healthy young adults [275], and with intestinal *Bacteroides* in healthy elderly women [276]. Thus, VO_2_max seems to be a good predictor of gut microbial diversity and metabolic function in healthy humans. Recent findings from the American Gut Project revealed that chronic exercise training benefits older adults by maintaining the stability of the gut microbiota (microbial composition and function) induced by aging [277]. Recent data demonstrated that masters athletes can be seen as a model of healthy aging also from the perspective of the microbiota [278]. Compared to community-dwelling older adults, senior orienteering athletes displayed a more homogeneous composition of gut microbiota, with higher levels of *F. prausnitzii* that is associated with positive health benefits, such as good gastrointestinal health, as well as psychological well-being that is most likely due to changes in the gut–brain axis [279]. Conversely, a substantial proportion of endurance athletes report gastrointestinal problems (e.g., abdominal discomfort, diarrhoea) during long-distance runs or competitive events and these symptoms may be related to gut ischaemia-associated leakage of bacterial endotoxins into the circulation [280]. Excessive exercise, but also other factors, such as drugs or illness, may be linked with dysbiosis of the gut microbiome, promoting inflammation and a catabolic state that can negatively influence muscle mass and function, particularly during aging [281].

Probiotics are live microorganisms that are thought to confer immunomodulation properties on both local and systemic immunity. Probiotics have been found to modify the population of the gut microflora and have been shown to increase some aspects of mucosal and systemic immunity in healthy humans such as altered cytokine production, increased natural killer cell cytotoxic activity, increased secretory immunoglobulin A (IgA) levels, and enhanced resistance to infections [282]. On the other hand, probiotics exert important anti-inflammatory ‘tolerogenic’ effects that may reduce the infection risk of athletes at a harmless level [258] and there is now some evidence from a number of studies in support of this [283,284,285,286,287].The proposed mechanism behind the protective effects of probiotics against infections in athletes may be attributable to modulation of the gut microbiota (enhanced intestinal barrier function and protection from pathogens) the mucosal immune system (enhanced bioactive metabolite production, such as SCFA and neurotransmitters) and lung macrophage and T lymphocyte functions [288,289]. Data from our own study indicate that some of these effects appeared to be connected with alterations in tryptophan metabolism [287]. Daily supplementation with a multi-species probiotics (1 × 10^10^ colony-forming units (CFUs) during 12 weeks of winter training limited exercise-induced drops in tryptophan levels and reduced the incidence of URI. Trained young athletes who developed URI demonstrated higher degradation rates of tryptophan compared to those without URI. A previous study undertaken by well-trained cyclists reported sex differences with probiotic supplementation (1 × 10^9^ CFUs of *L. fermentum* PCC^®^) for 11 weeks with a significant reduction in lower respiratory illness symptoms (duration and severity) in males, but with some evidence of an increase in symptoms in females, while the effects of probiotic supplementation on URI were unclear in males and females [290]. Although anti-inflammatory effects of probiotics exist in young athletes, these results cannot be directly extrapolated to masters athletes due to changes in the immune system that occur with ageing. The influence of probiotics on immune function and infection risk in the elderly has been studied only sporadically. The most comprehensive study so far in free-living elderly aged >65 years demonstrated a clear association between probiotic (3 × 10^7^ CFUs of *L. delbrueckii* ssp. *bulgaricus* 8481) consumption for 6 months and enhanced systemic immunity in older people [291]. Clinical benefits by probiotics are reported from respiratory infection studies where in one study the rate of common cold infections was decreased by dietary intake of yoghurt fermented with *L. delbrueckii* ssp. *bulgaricus* OLL1073R-1 [292] and in another study the duration but not the incidence of episodes decreased with *L. casei* DN-114001 [293]. Theoretically, combining probiotics with omega-3 fatty acids may offer a promising nutritional strategy to counteract metabolic challenges associated with aging via the gut microbiota, especially relevant for older people that suffer from anabolic resistance, systemic inflammation, and mood disorders (through the gut–brain axis) [279,294,295]. However, more research is required to understand the connection between exercise, nutrition and the gut microbiota in the elderly population in general and in master athletes in particular.

## 5. Conclusions and Future Perspectives

**Figure 6:**
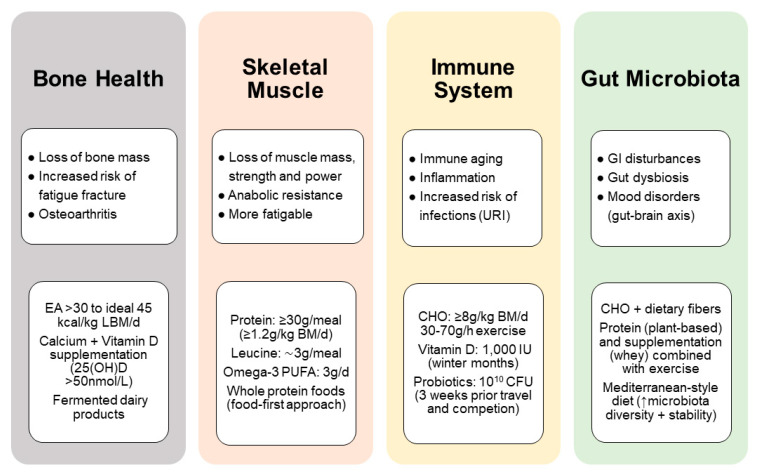
Overview of physiological and diet-related challenges associated with aging and nutritional strategies to assist masters athletes to stay healthy, optimize adaptation and post-exercise recovery. Body mass, BM; colony-forming units, CFU; carbohydrates, CHO; energy availability, EA; gastrointestinal, GI; international units, IU; lean body mass, LBM; polyunsaturated fatty acids, PUFA; upper respiratory illness, URI.

In summary, physiological and diet-related challenges that occur with the aging process can be positively influenced by chronic exercise training and appropriate nutrition. Even though limited evidence exists on nutritional requirements for masters athletes, based on the analysis of current nutrition guidelines for young athletes and older non-athletic populations, recommendations can be suggested for older athletes (Figure 6). Particular attention should be given to proper energy and protein intake for preventing low bone and muscle mass and optimizing post-exercise recovery. Furthermore, a higher demand for specific nutrients (e.g., vitamin D, probiotics omega-3 fatty acids) should be considered in older athletes to improve both mucosal and systemic immunity, enhance resistance towards inflammatory and metabolic diseases (including infections), and, last but not least, to preserve psychological well-being, which itself can help boost the masters athletes’ immune system.

Although we have proposed nutritional recommendations for older athletes, there are still many open questions concerning the nutritional requirements of masters athletes. For example, how do older athletes train and eat and, what is the nutrition knowledge and practice of masters athletes? What role does the diet play, for both decreasing fracture risk and enhancing the healing process after fracture? In addition, further research is required demonstrating the benefits of tolerogenic nutrients for their immunological effects in older athletes. There is limited research available on how adaptations to exercise impact the gut microbiota and how nutritional factors, such as restricted energy or higher protein consumption, influence the gut microbiota in older athletes. As such, future longitudinal studies should address what kind of diet and specific nutrients are most suitable for the athletic lifespan to better assist masters athletes.

## Data Availability

Data sharing is not applicable to this article.
